# Investigating organic multilayers by spectroscopic ellipsometry: specific and non-specific interactions of polyhistidine with NTA self-assembled monolayers

**DOI:** 10.3762/bjnano.7.48

**Published:** 2016-04-13

**Authors:** Ilaria Solano, Pietro Parisse, Ornella Cavalleri, Federico Gramazio, Loredana Casalis, Maurizio Canepa

**Affiliations:** 1 Dipartimento di Fisica, Università di Genova, Via Dodecaneso 33, Genova, Italy; 2 Elettra Sincrotrone Trieste S.C.p.A., s.s. 14 km 163,5 in Area Science Park, Basovizza, Trieste, Italy

**Keywords:** His-tag, nitrilotriacetic acid (NTA), protein binding, self-assembled monolayers (SAMs), spectroscopic ellipsometry

## Abstract

**Background:** A versatile strategy for protein–surface coupling in biochips exploits the affinity for polyhistidine of the nitrilotriacetic acid (NTA) group loaded with Ni(II). Methods based on optical reflectivity measurements such as spectroscopic ellipsometry (SE) allow for label-free, non-invasive monitoring of molecule adsorption/desorption at surfaces.

**Results:** This paper describes a SE study about the interaction of hexahistidine (His_6_) on gold substrates functionalized with a thiolate self-assembled monolayer bearing the NTA end group. By systematically applying the difference spectra method, which emphasizes the small changes of the ellipsometry spectral response upon the nanoscale thickening/thinning of the molecular film, we characterized different steps of the process such as the NTA-functionalization of Au, the adsorption of the His_6_ layer and its eventual displacement after reaction with competitive ligands. The films were investigated in liquid, and ex situ in ambient air. The SE investigation has been complemented by AFM measurements based on nanolithography methods (nanografting mode).

**Conclusion:** Our approach to the SE data, exploiting the full spectroscopic potential of the method and basic optical models, was able to provide a picture of the variation of the film thickness along the process. The combination of δΔ*_i_*_+1_*_,i_*(λ), δΨ*_i_*_+1_*_,i_*(λ) (layer-addition mode) and δΔ^†^*_i_*_',_*_i_*_+1_(λ), δΨ^†^*_i_*_',_*_i_*_+1_(λ) (layer-removal mode) difference spectra allowed us to clearly disentangle the adsorption of His_6_ on the Ni-free NTA layer, due to non specific interactions, from the formation of a neatly thicker His_6_ film induced by the Ni(II)-loading of the NTA SAM.

## Introduction

The design of biosensing devices requires precise immobilization, with controlled orientation, of functional proteins on a surface, generally consisting of a substrate functionalized with a molecular receptor layer [1–2].

After seminal works [3], the interaction of chelated Ni ions with oligohistidine peptides has been largely implemented over the years for the affinity purification of recombinant proteins [4–6], and for the development of bioanalytical surfaces [7–12]. To this purpose, self-assembled monolayers (SAMs) terminated with the nitriloacetic acid (NTA) group, after loading with nickel ions (Ni(II)), provide platforms able to specifically bind his-tag proteins [13–18] and enzymes that retain their activity upon immobilization [19–21]. The affinity between the adsorbent surface and the protein can be modulated, e.g., by using multi-chelator constructs [22–23] or double hexahistidine tags [24].

Among applications, the NTA–Ni(II)–His coupling has been exploited for the functionalization of nanoparticles [25–27] and atomic force microscopy (AFM) tips [28] as well as for more biologically oriented applications such as the delivery of polymeric gene vehicles [29] or for the design of NTA-functionalized fluorescent probes able to track cellular events in situ by targeting specific His-tagged proteins [30–31].

Beyond selectivity, the reversibility of the NTA–Ni(II)-His coupling upon reaction with competitive ligands [32] makes this strategy a versatile system for the development of highly sensitive, specific and renewable sensing devices [33].

A high-performance sensor needs to integrate a specific binding scheme with a sensitive, non-destructive transduction of the binding event into a measurable signal. Optical methods based on optical reflectivity, such as surface plasmon resonance (SPR) and ellipsometry, allow for label-free, non-invasive, in situ monitoring of molecular adsorption/desorption at surfaces [34–40]. While SPR-based methods require supports with specific plasmonic properties, ellipsometry methods can be employed on a variety of reflecting solid surfaces. Spectroscopic ellipsometry (SE) can detect molecular adsorption on the receptor layer through the observation of thickness variations and, more specifically, through the spectroscopic characterization of UV–vis absorptions related to the analyte or to the receptor–analyte bond.

At variance with more traditional fields of application of SE [41–43], a reliable analysis of SE data on ultrathin films, at the nanometer scale thickness, must rely on the evaluation of the fine changes of the ellipsometry response upon the thickening/thinning of the molecular film. Such variations can be emphasized looking at so-called difference spectra [44], δΨ*_i_*_+1_*_,i_*(λ), δΔ*_i_*_+1_*_,i_*(λ), where the suffix labels the difference between the spectra taken after and before the addition of the (*i* + 1)-th layer in a multilayer stack.

Our approach, developed in recent years [45–46], aims to exploit, under an unconventional perspective, the full spectroscopic potential of difference spectra. Regarding the important class of thiolate SAMs on flat gold films, careful analysis of δ-spectra allowed us to disentangle spectral features related to the SAM and to the S–Au interface region [46] as well as to identify fingerprints of molecular UV–vis absorptions [47–48], with sub-monolayer sensitivity.

In a very recent work we successfully combined our approach with thickness determination based on AFM measurements exploiting smart nanolithography methods, such as the so-called nanoshaving [49], that considerably helped to reduce the uncertainty related to the correlation between thickness and refractive index that is typical of ultrathin films.

**Figure 1:**
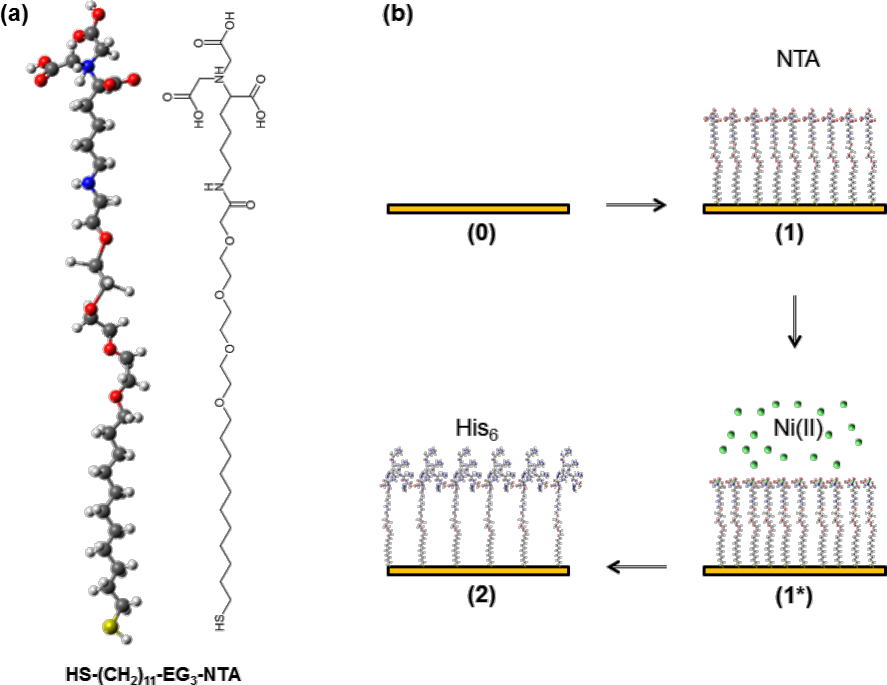
(a) Chemical structure of the HS-C11-EG3-NTA (NTA1) molecule; (b) sketch of the steps of sample preparation: (0) substrate preparation, (1) formation of the NTA SAM, (1^*^) Ni(II) loading and (2) adsorption of His_6_. Step (1^*^) was skipped for a subset of samples in order to check non-specific His_6_–NTA interactions. The ellipsometry analysis focused on steps (0), (1), (2).

A step forward, we intend to apply the same methods to control the adsorption of the analyte layer (AL) on the receptor layer (RL), eventually distinguishing between specific and non-specific binding. In this article we describe a study about the interaction of His_6_ on gold substrates functionalized with NTA (Figure 1). While on simple NTA-terminated films only non-specific adsorption of His_6_ is expected, the loading with Ni(II) endows the RL with specific affinity towards His_6_. We investigated the sequence of steps of the process (schematically sketched in Figure 1) beginning from the functionalization of Au until the final regeneration of the NTA layer through displacement of His_6_. To this aim we will introduce convenient 
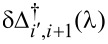
 and 
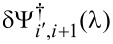
 difference spectra where the suffix labels the difference between the spectra taken after and before the removal of the (*i* + 1)-th layer in a multilayer stack.

The systems were investigated in situ in liquid, and ex situ in ambient air. The experiments aimed to explore the variation of film thickness after convenient stages of the process as well as to detect spectral features that could be eventually related to the specific NTA–Ni(II)–His_6_ interaction. The SE investigation has been usefully complemented by AFM measurements based on SAM nanolitography (both shaving and grafting modes).

## Results and Discussion

### Substrate functionalization: the NTA-SAM

**Figure 2:**
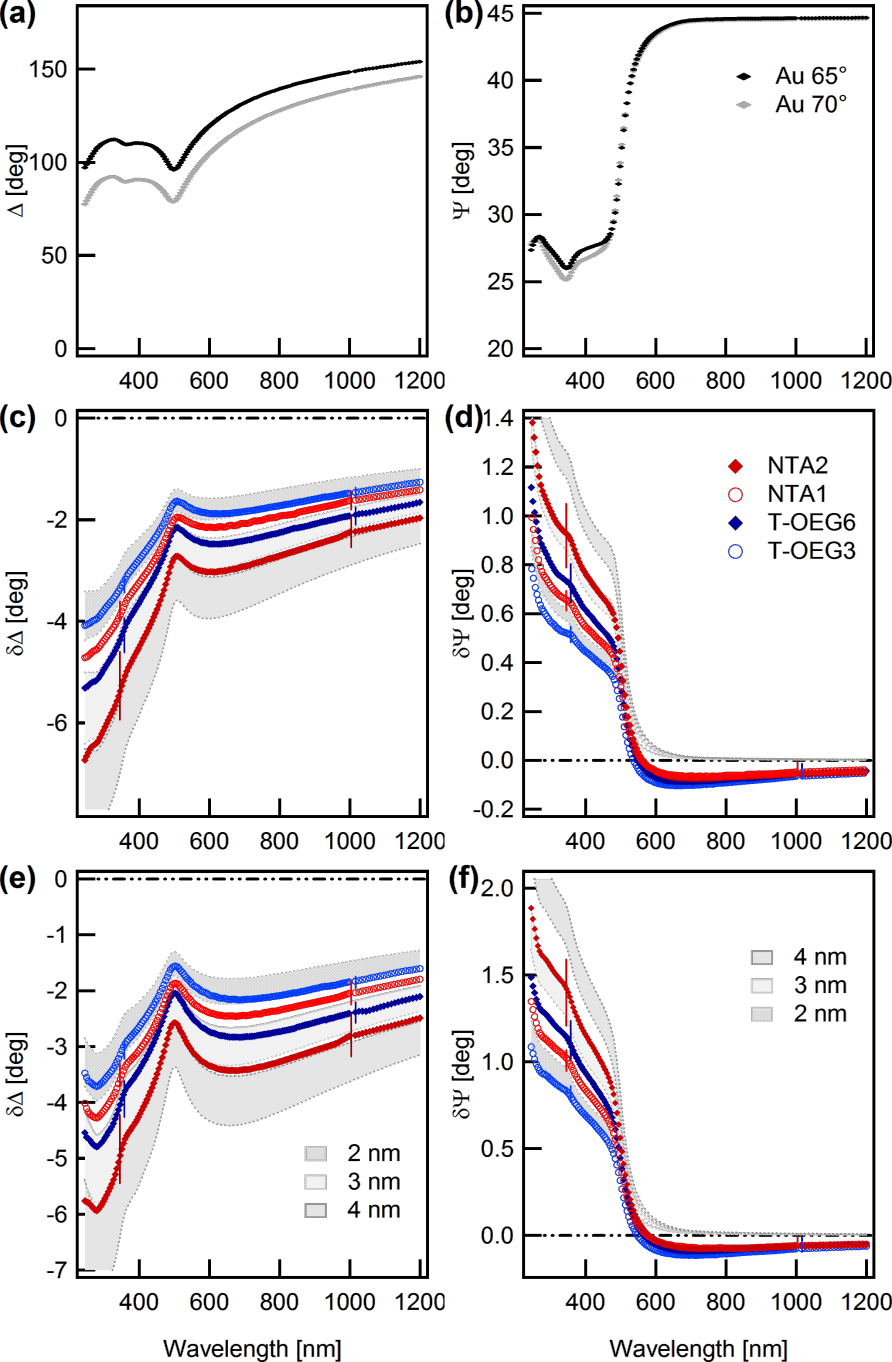
(a,b) Ex situ experimental SE spectra obtained on gold substrates. (c–f) Ex situ experimental difference spectra (δΔ_1,0_ = Δ_1_ − Δ_0_, δΨ_1,0_ = Ψ_1_ − Ψ_0_) obtained for NTA2 (red full diamonds) and NTA1 (red open circles) SAMs. Panels c and d: 65° angle of incidence, panels e and f: 70° angle of incidence. Data from T-OEG6 and T-OEG3 SAMs [49] are also shown for reference. Thin vertical bars represent the experimental uncertainty related to data dispersion over the investigated samples. Shaded areas illustrate the results of simulations based on the TF|Au model, for three values of the SAM thickness (*d*_TF_ = 2, 3 and 4 nm). For δΔ_1,0_ patterns (left) the top and bottom borders of the shadings correspond to *n*_TF_ = 1.35 and 1.50, respectively. For the δΨ_1,0_ spectra (right panels) the order is reversed.

The first step of the experiment consisted of the formation and the characterization of the NTA-terminated precursor SAMs. Details on the preparation are reported in the Experimental section. Note that an accurate characterization of the precursor receptor SAM is a necessary prerequisite for a reliable interpretation of measurements about adsorption of analytes. Ex situ SE δΔ_1,0_ and δΨ_1,0_ spectra are presented in Figure 2 for two types of SAMs (HS-C11-EG3-NTA, NTA1, and HS-C16-EG3-NTA, NTA2). The suffixes 1 and 0 of δΔ_1,0_ and δΨ_1,0_ correspond to the SAM–Au and to the Au system, respectively. The results from NTA SAMs are compared with those obtained recently for oligo(ethylene glycol)-terminated alkanethiols, HS-C11-EG3 (T-OEG3) and HS-C11-EG6 (T-OEG6), for which the thicknesses were accurately determined by SE and AFM nanolithography [49].

The shape of the NTA spectra closely resembles that of T-OEG SAMS and does not show any evident features typical of intrinsic molecular optical absorptions [47,50]. Thus, the data are presented in comparison with a set of simulations based on the model of a transparent film (TF) onto the substrate (Au), assuming for TF a Cauchy-type dispersion formula [42]:

[1]
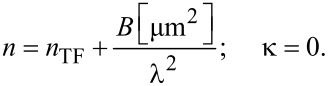


The shaded areas in Figure 2 span the representative range of 1.35 ≤ *n*_TF_ ≤ 1.50 [51], for three indicative values of the film thickness *d*_TF_. The coefficient *B* was set to the value found in the analysis of T-OEG molecules (0.005) [49]. Note that the extension of the shaded bands provide an intuitive picture of the well-known correlation between refractive index and thickness that is typical for ultrathin films.

Systematic studies carried out in our group and, in particular, the analysis of the TF|Au model performed in [49] showed that δΔ_1,0_ and δΨ_1,0_ spectra provide a somewhat complementary information. While the δΔ_1,0_ spectra are sensitive to the properties of film + interface throughout the whole spectral range, the δΨ_1,0_ spectra show an interesting behavior in connection with the high reflectivity onset of Au which is evident in Ψ spectra (Figure 2b) between 500 and 550 nm.

Above this threshold, going towards the NIR, the TF|Au model predicts that Ψ tends to assume values close to 45° insensitive to transparent films: Simulated δΨ_1,0_ values vanish. Negative experimental δΨ_1,0_ values are due only to the absorptive properties of the interface layer, in connection with the formation of a strong molecule–gold bond [46,52].

In contrast, below the threshold, δΨ_1,0_ spectra bear substantial information only about the transparent part of the film [49]. Further, compared to the δΔ_1,0_ spectra, δΨ_1,0_ spectra are less affected by the correlation between *n*_TF_ and *d*_TF_.

Thus, from careful inspection of the δΨ_1,0_ patterns in Figure 2, and considering the uncertainties of experiment and model, one can estimate *d*_TF_ values of 2.2 ± 0.3 and 3.1 ± 0.3 nm for the NTA1 and NTA2 SAMs, respectively. Negative δΨ_1,0_ values, which are substantially common to all the SAMs of Figure 2 and closely comparable to other well-structured thiolate–Au SAMs [46–47,53–54], can be effectively reproduced by simulations considering an effective interface layer [55] with effective thickness *d*_I_ of the order of 0.2 nm [46,49]. The sum *D* of *d*_I_ and *d*_TF_ (2.4 ± 0.3 and 3.3 ± 0.3 nm for the NTA1 and NTA2 SAMs, respectively) accounts for the total film thickness that is reflected in the δΔ_1,0_ spectra.

**Figure 3:**
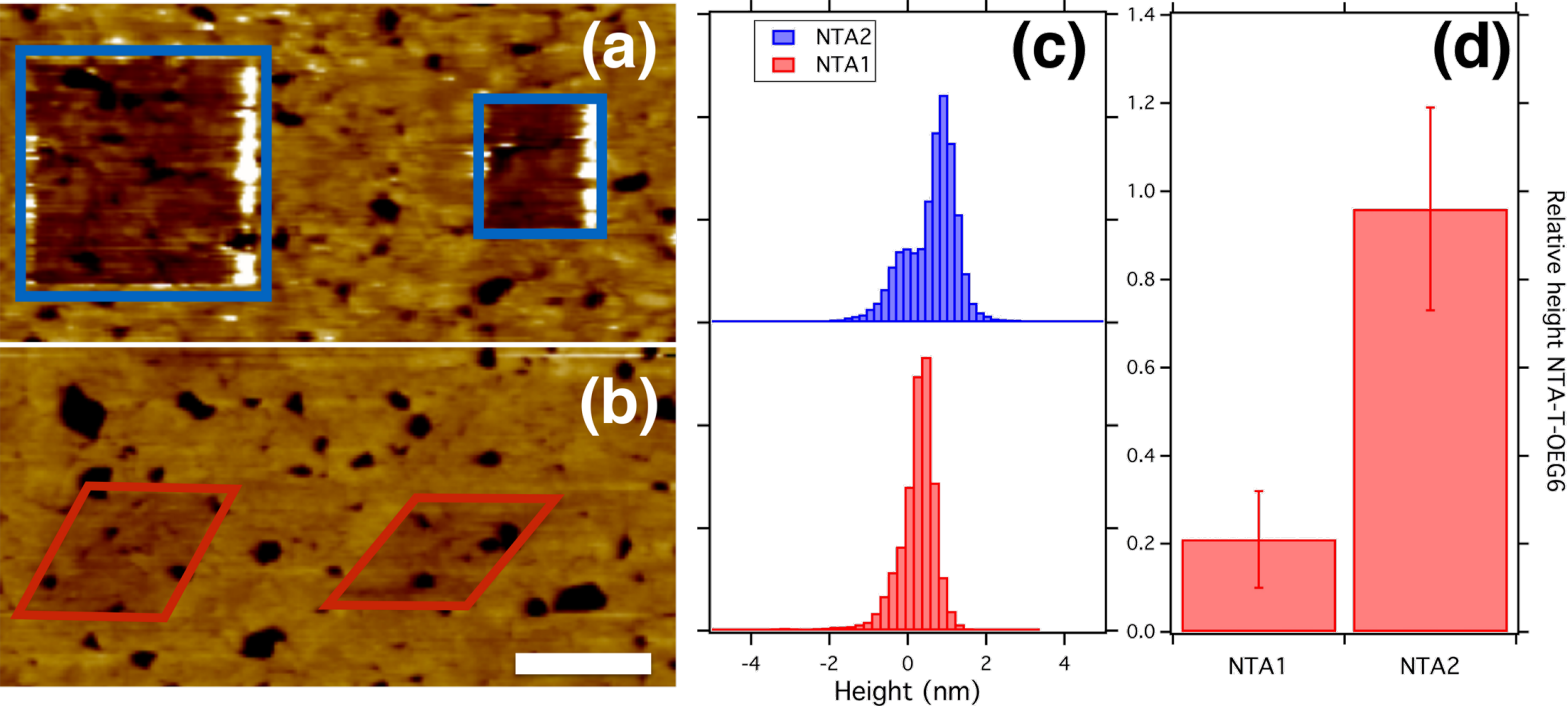
(a) Atomic force microscopy image showing two nanografted patches of T-OEG6 in a NTA2 SAM (b) the same for a NTA1 SAM. The white scale bar corresponds to 1 μm. (c) Height histograms corresponding to the AFM images in (a) and (b). The reference has been set at the center of the Gaussian distribution related to the squared dark areas in the images. (d) Synthesis of histogram analysis for the NTA1 and NTA2 samples investigated providing the average values of Δ*h*_NTA−TOEG6_ and Δ*h*_NTA1−TOEG6_ (for details see the text).

The AFM images of Figure 3a are representative of experiments of nanografting of T-OEG6 molecules in NTA SAMs. The latter were deposited with the same procedures applied in the SE measurements. The grafted areas are darker than the surrounding SAM carpet, indicating a lower height level. The histograms reported in Figure 3c show distributions that can be fitted by the superposition of two Gaussian curves. One, related to the T-OEG6 surface is taken as the reference; the second corresponds to the surface of the NTA SAM. The separation between the centers of the Gaussian curves provides Δ*h*_NTA−TOEG6_, the height difference between the NTA and T-OEG6 SAMs.

After repeating this procedure for ten grafted patches we were able to obtain an average value of Δ*h*_NTA2−TOEG6_ of 0.95 ± 0.15 nm (Figure 3d). Regarding NTA1 SAMs, the contrast with the nanografted T-OEG6 patches is much lower (Figure 3b). In this case the analysis of images provides Δ*h*_NTA1−TOEG6_ of 0.2 ± 0.1 nm (Figure 3d). Combining these findings with the AFM results regarding the height of T-OEG6 SAMs presented in [49], which amounted to 2.4 ± 0.3 nm, we obtain for the NTA1 and NTA2 films an AFM thickness of 2.6 ± 0.4 and 3.4 ± 0.5 nm, respectively.

The height difference between NTA2 and NTA1 SAMs measured with AFM (Δ*h*_NTA2−NTA1_ = 0.8 nm) and the thickness difference obtained by SE (Δ*D*_NTA2−NTA1_ = 0.9 nm) agree with the nominal length difference between the two molecules, of about 0.8 nm. However, although AFM and SE findings are compatible within the experimental uncertainty, the measurements by AFM yield height values that are systematically larger than the ellipsometry thickness. Small discrepancies between the two methods appear conceivable and even expected for several reasons. First of all, we need to consider that the NTA head is more charged than the OEG termination and can be responsible of an increased electrostatic repulsion and therefore of an increased height during the measurements in liquid. Moreover, we should consider systematic differences regarding the macroscopic area probed by the optical beam in SE vs the microscopic area probed by AFM and the different environment of the measurements (liquid for AFM, air for SE).

Note that SE does not provide direct information about molecular orientations in the SAM and about the SAM organization. Such properties should be probed through surface-sensitive infrared spectroscopy methods such as reflection–absorption infrared spectroscopy [56]. Nevertheless, the values of film thickness determined by SE and AFM testify the formation of compact NTA SAMs, which can be used for second-layer adsorption experiments.

### His_6_–NTA interaction

Exploratory checks showed that the variations induced by Ni(II) loading on the NTA/Au SE spectra were very small. Thus, we decided to study the His_6_ adsorption (step (2) in Figure 1) by referencing the difference spectra to the data of the NTA film. Specific or non-specific interactions of His_6_ are therefore probed through a comparison of the results obtained with or without the Ni(II) pre-loading. We adopted preparation protocols described in literature [57], which are summarized in the Experimental section.

**Figure 4:**
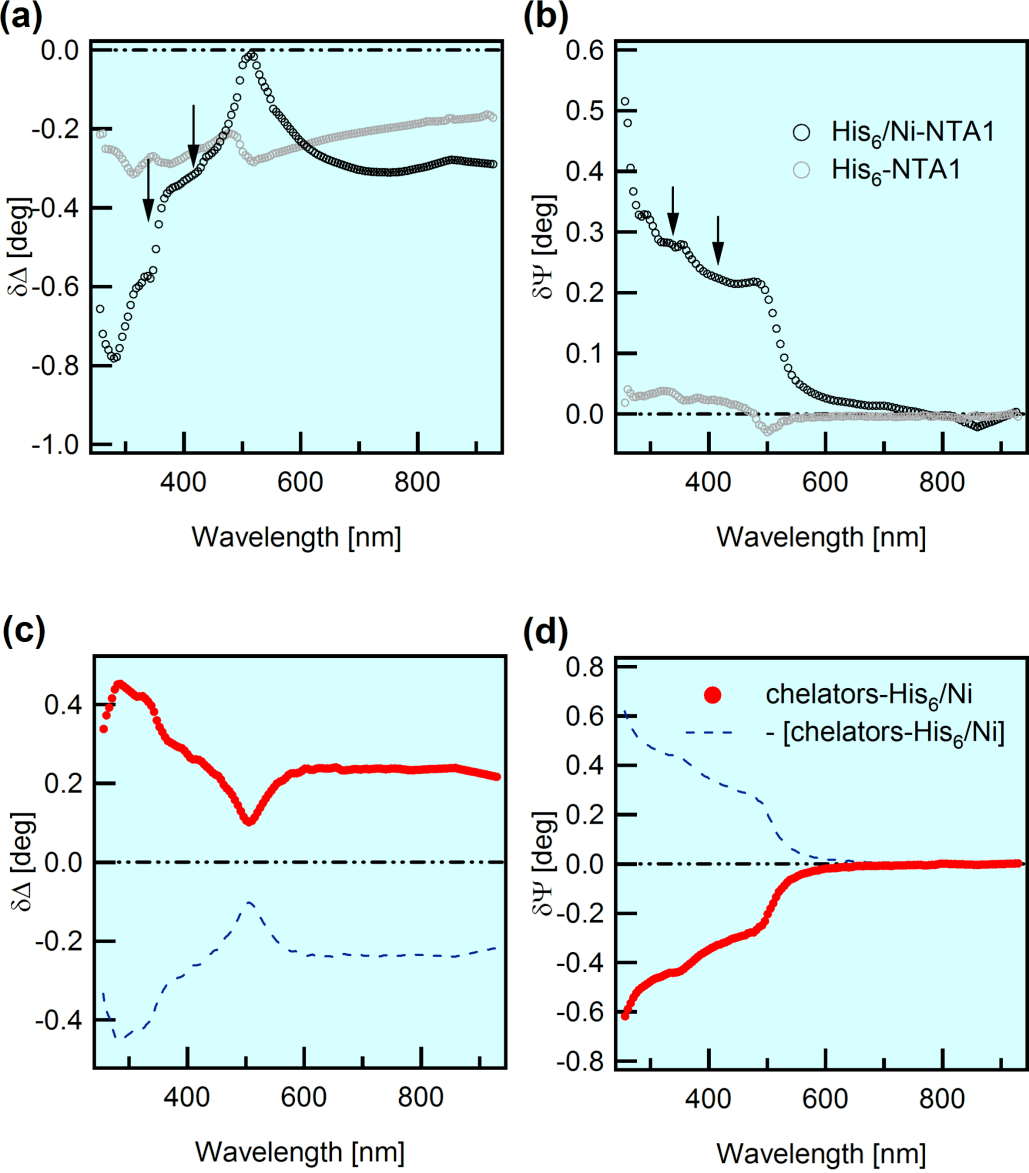
His_6_ adsorption on the NTA/Au system and subsequent treatment with imidazole/EDTA. The SE data are derived from in situ (buffer) measurements at 65° angle of incidence. (a,b) δΔ_2,1_ and δΨ_2,1_ difference spectra; the suffixes 1 and 2 indicate the NTA1/Au system before and after the exposure to His_6_ molecules, respectively. Black and light grey symbols refer to measurements with and without Ni(II) loading, respectively. Arrows indicate possible weak optical absorptions (see the text for details). (c,d) Red symbols: 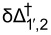, 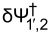 spectra; the suffixes 1’ and 2 stand for the His_6_/NTA1/Au system after and before the treatment with chelating agents. The blue dashed curves represent 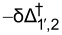 and 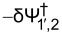 spectra.

δΔ_2,1_ and δΨ_2,1_ difference spectra shown in Figure 4a,b are representative of the observed behavior. Despite the reduced refractive index mismatch between the film and the ambient and the use of cell windows tending to lower the signal-to-noise ratio, the emerging picture appears rather sharp.

In the case of Ni-free RL (light grey circles) we found very low δΨ_2,1_ and δΔ_2,1_ spectral values, over the whole wavelength range. These results suggest a very modest thickness increment. In facts, the formation of films uniformly spread on gold-like substrates, even if very thin, usually induces a well-defined relative maximum of δΔ*_i_*_+1_*_,i_* at about 500 nm [46,52]. This is actually missing in the data, possibly indicating the formation of a low-coverage sub-monolayer [58], a situation that is compatible with non-specific His_6_–NTA interactions, in accordance with literature [10].

Conversely, well-structured spectra were observed after the interaction of His_6_ with Ni-loaded NTA SAMs (black open circles), testifying the formation of a well-defined layer. Note in passing that Ni(II) loading and subsequent His_6_ adsorption have very little influence on the δΨ_2,1_ values above 550 nm, which are overall positive and close to zero. This finding suggests that the percolation of Ni through the compact NTA SAM down to the S–Au interface is likely negligible.

In order to add confidence about the formation of the Ni(II)–His_6_ complex, we analyzed its interaction with competitive chelating agents eventually leading to the regeneration of the NTA film. Representative difference between spectra measured after and before the imidazole/EDTA treatment, for brevity 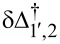 and 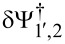, are shown in Figure 4c,d (red open circles).

The graphic choice in panels c and d emphasizes that 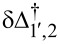 and 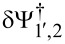 spectra are specular (with respect to the null level) to δΔ_2,1_ and δΨ_2,1_ spectra which have been considered until now (and in all our previous works on SAMs on Au). δΔ*_i_*_+1_*_,i_* and δΨ*_i_*_+1_*_,i_*, were always associated with the addition of a molecular layer (for example the NTA layer on the Au substrate or the His_6_ layer on the NTA/Au system). In the case of Figure 4c,d, positive 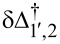 and low-wavelength, negative 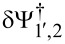 (red curves) indicate a neat decrease of the thickness, to be associated with the removal of molecular layer 2 (His_6_).

**Figure 5:**
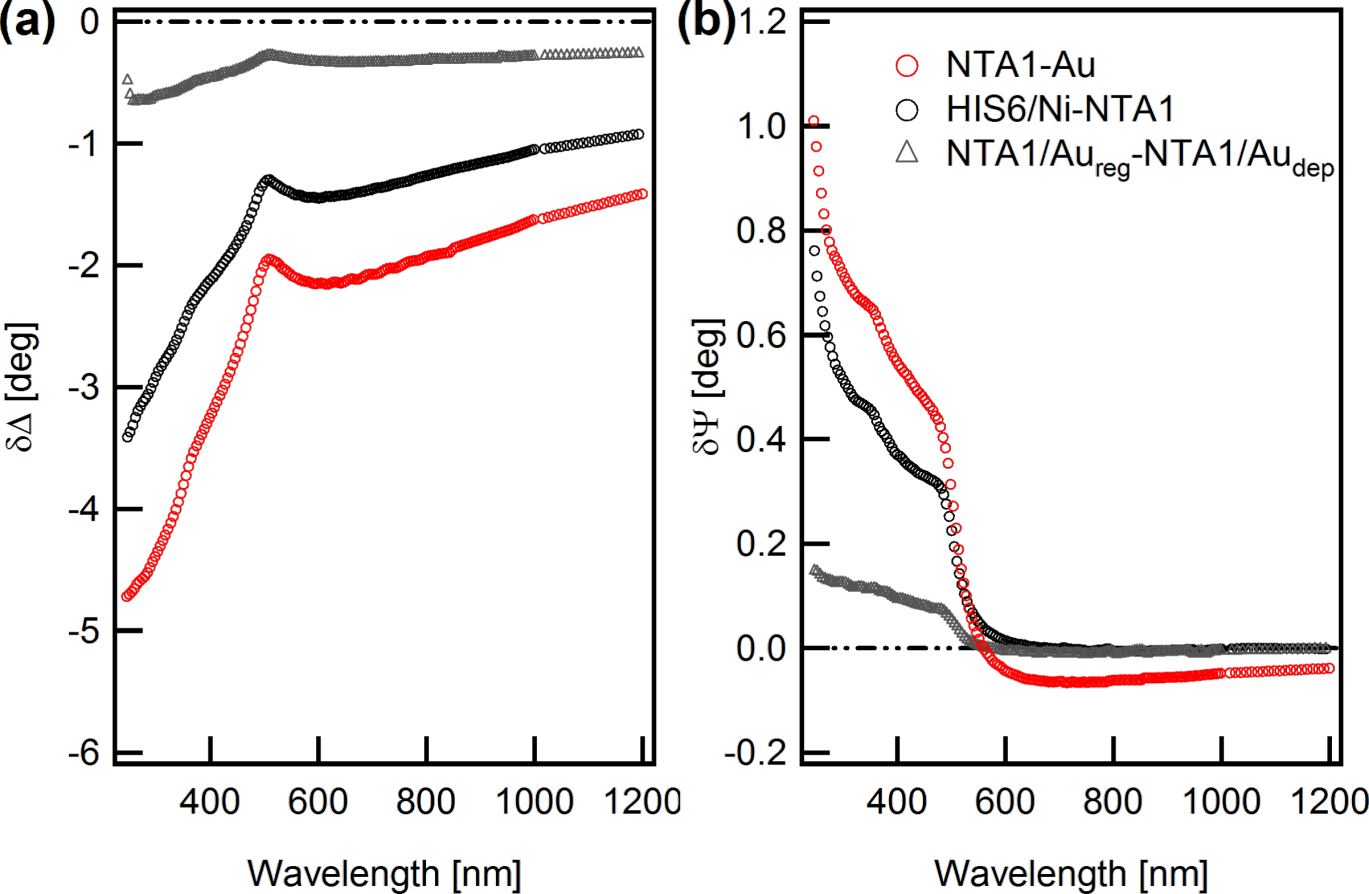
δΔ_2,1_ and δΨ_2,1_ spectra (black circles), related to the His_6_ adsorption, are compared with δΔ_1',1_ and δΨ_1',1_ spectra (grey triangles) obtained after the completion of the imidazole/EDTA treatment. These difference spectra are referenced to the NTA/Au system. δΔ_1,0_ and δΨ_1,0_ spectra associated to the deposition of NTA1 on the Au substrate (red circles) are also reported for comparison.

Further insight into thickening or thinning of the films following specific adsorption and removal of His_6_ was obtained from ex situ measurements. In Figure 5, δΔ_2,1_ and δΨ_2,1_ spectra (black symbols), related to the His_6_ adsorption, are compared with δΔ_1',1_ and δΨ_1',1_ spectra (grey symbols) obtained after the completion of the imidazole/EDTA treatment. Note that these difference spectra are referenced to the NTA/Au film. In the figure, we also report the δΔ_1,0_ and δΨ_1,0_ spectra from Figure 2, referenced to the bare gold substrate. Ideally, δΔ_1',1_ and δΨ_1',1_ should present values close to the zero. Indeed, they appear significantly lower than the δΔ_2,1_ and δΨ_2,1_ spectra, indicating the thinning of the film.

The ex situ measurements are also useful for an estimate of the second layer thickness exploiting its rough proportionality with the NIR δΔ_2,1_ values, considering the NTA/Au film as reference and performing the same type of analysis as that in Figure 2. With a reasonable guess for the His_6_ layer refraction index in the range of 1.35–1.40, the layer thickness is estimated in the range of 1.5–1.7 nm range. Analogously, we can also estimate that the imidazole/EDTA treatment removed ≈75% of the His_6_ layer. The removal percentage could be even higher considering the conceivable effect of imidazole/EDTA residuals in δΔ_1',1_ and δΨ_1',1_ spectra. Therefore, the SE analysis of both in situ and ex situ data is able to follow the thickness variations induced by the addition/removal of the Ni(II)–His_6_ layer.

An important step further would be the detection of spectral fingerprints of the Ni(II)–His_6_ bond. In this respect, a comprehensive transmission spectroscopy study in solution reported optical absorptions of the Ni(II)–His_6_ complex in the spectral range of 300–700 nm. Shape and intensity of absorption bands were found to depend on the Ni(II)/His_6_ concentration ratio and the pH value of the solution [15]. Indeed, in previous SE works on other SAMs on gold, we could detect optical molecular fingerprints as characteristic narrow-band dips in difference spectra. Specific examples are the intense Soret band observed in in situ spectra of cytochrome C SAMs [48,50] or the UV absorptions of carbazole groups [47]. Detection of molecular fingerprints becomes definitely easier as the thickness scales up, as in the case of a 30 nm thick photochromic polymer film [59].

Due to index matching issues, observation of optical absorptions in SAMs becomes easier in liquid with refractive index close to that of the film [48,50]. A close look at in situ data (Figure 4) shows fine spectral features (indicated by arrows) which cannot be reproduced by a simple Cauchy model, and could be tentatively attributed to optical absorptions. However, these features are not intense and sharp enough for an unambiguous assignment, which would require, e.g., a check of K–K consistency, as it was possible in previous experiments on other molecular systems [48,50,59]. Therefore the direct spectroscopic detection of the Ni(II)–His_6_ compound is still uncertain and will require further investigation.

## Conclusion

We have applied spectroscopic ellipsometry to investigate the interaction of His_6_ with gold surfaces functionalized with a thiolate, OEG SAM bearing the NTA end-group, a system which is of recognized relevance for the design of a whole class of bio-sensing devices.

Indeed, ellipsometry was already employed to study related systems. In [37], about mixed layers of bis-NTA thiols with inert OEG thiols, the authors thoroughly reported on imaging ellipsometry measurements at a single wavelength. They exploited variations of Δ and Ψ to obtain information about the thickness and homogeneity of the mixed layers while their functionalities were probed using imaging SPR. In [51] the NTA–Ni(II)-mediated immobilization of His_6_-tagged green fluorescent protein on a silica surface was reported. Ellipsometry, at two wavelengths, was exploited mainly to characterize the precursor NTA SAM (about 15 nm thick) while the interactions with proteins of the NTA surface were studied by fluorescence methods. Spectroscopic ellipsometry was also frequently used on other NTA-related systems as an ancillary characterization method [10,60–61].

To our knowledge this is the first time that SE has been used to comprehensively address the process, from the characterization of the precursor NTA layer to the study of the Ni(II)-mediated His_6_ absorption and the final regeneration of the NTA layer obtained by exposure to competitive chelating agents.

Our approach, progressively developed in recent years, is based on the careful analysis of SE difference spectra bearing information on the small changes of the ellipsometry response upon the thickening or thinning of molecular films with nanoscale thickness, and aims to fully exploit the rich broadband potential of SE, a point which marks the difference with single wavelength techniques, including SPR. Difference spectra, measured for hundreds of wavelengths ranging from the short UV to the NIR, proved able to show the variation of film thickness related to a complex process consisting of addition and removal of several organic layers.

Our investigation addressed the characterization of the NTA SAM on gold, exploiting the powerful cross-check with AFM measurements of the SAM height based on smart nanolitography methods. The SE data, which have been interpreted with basic models, provided a reliable evaluation of the thickness and optical properties of compact NTA SAMs. The accurate knowledge of the properties of the functionalizing SAMs constituted the necessary base for the second step of the experiment which regarded the investigation of the NTA reaction with His_6_. The experiments were not conclusive regarding the direct spectroscopic detection of the NTA–Ni(II)–His_6_ bond, which will deserve further investigation. Nonetheless, the combination of δΔ,Ψ*_i_*_+1_*_,i_*(λ) (layer-addition mode) and 
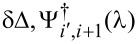
 (layer-removal mode) allowed us to clearly disentangle the formation of a His_6_ film induced by Ni(II)-loading of the NTA SAM from the non-specific adsorption of His_6_ on the Ni-free NTA layer.

This investigation is intended to form a solid analytical background towards the study of layered systems of greater complexity such as the interaction of NTA with His_6_-tag proteins, or the DNA/DNA hybridization, which is ongoing in our laboratories. Further, this paper describes a quite general method, which can be applied to other forms of organized organic nano-multi layers on solid as well liquid reflecting substrates.

## Experimental

### Gold substrates and reagents

Gold films for optical measurements were purchased from Arrandee^TM^ (200 nm thick, deposited on glass with a Cr primer). For AFM measurements Ulman-type [62] ultra-flat stripped gold surfaces were preferred. Ultra-flat gold samples were prepared by UHV deposition of gold onto a freshly cleaned Si(111) wafer. A silicon sample was then glued on the gold surface and cured overnight at 130 °C. Ultraflat and clean gold surfaces are obtained by mechanically stripping the silicon wafer substrate from the silicon gold sandwich [49,62].

(1-Mercaptoundec-11-yl)tri(ethylene glycol) and (1-mercaptoundec-16-yl)tri(ethylene glycol) with NTA (for brevity NTA1 and NTA2) were purchased from Prochimia Surfaces. Hexahistidine His_6_ (840.85 g/mol) was purchased from GenScript. Ethylenediaminetetraacetic acid (EDTA), imidazole, tris(hydroxymethyl)aminomethane (Tris), NaCl and NiCl_2_ were purchased from Sigma-Aldrich.

### NTA SAM deposition and interaction with His_6_

Clean Arrandee samples or freshly stripped Ulman gold samples were incubated for 24 h in 15 μM NTA1 (NTA2) solution, using ethanol as solvent. For ex situ optical measurements the NTA/Au samples were rinsed with ethanol and dried with N_2_ flow. A subset of NTA/Au samples were transferred in a salt buffer solution (20 mM Tris and 150 mM NaCl, pH 7.4) for subsequent characterization and treatments. After a first optical characterization in the new medium, the samples were immersed in 0.5 M EDTA (pH 8.6) solution for 15 min in order to remove undesired divalent metal ions and rinsed in MilliQ water. Soaking the NTA samples in a solution of 10 mM NiCl_2_ in 20 mM Tris (pH 7.4) for 5–10 min allowed for the coordination of NTA COOH groups with Ni^2+^ ions. The samples were then immersed in the salt buffer solution containing a 170 μM concentration of His_6_. A subsets of samples were exposed to His_6_ without pre-emptive Ni loading. Finally the samples were rinsed with the buffer solution, characterized by SE in situ, rinsed in MilliQ water, fluxed with N_2_ and characterized by SE ex situ. Regeneration of the NTA/Au surface was accomplished by rinsing the sample in 0.4 M imidazole in 20 mM Tris solution and then in 0.5 M EDTA (pH 8.6) solution.

### Spectroscopic ellipsometry

SE measurements were performed on a rotating compensator instrument (M-2000, J.A.Woollam Co.Inc.). Ex situ data were collected at 65–70° angle of incidence. The presented spectra were obtained after the averaging over five samples and over four different zones of each sample. In situ measurements were conducted in a custom-made Teflon cell with 65° angle of incidence. The in situ data are presented in the transparency region of the solvent. Principles and applications of SE are detailed in books [42–43] and reviews [63–64]. Briefly, standard ellipsometry consists in the investigation of the coefficient 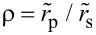 = tan Ψ e*^i^*^Δ^ where 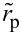 and 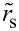 are the complex Fresnel reflection coefficients related respectively to p- and s-polarization. The ellipsometric analysis is performed through comparison between experimental and simulated data. Simulations are based on models that take into account optical and morphological properties of the layered sample.

### AFM nanografting and imaging

AFM experiments were carried out with a XE100 (Park Instruments) instrument in custom liquid cells. For nanografting, the freshly prepared NTA SAM samples were glued in a custom liquid cell filled with a solution of 30 μM HS-C11-OEG6 (T-OEG6) in ethanol. During the nanografting process, we operated in contact mode with commercial AFM tips (NSC19 Mikromasch, *k* = 0.6 N/m), applying a high load (100 nN) in a confined (from 0.5 × 0.5 to 2 × 2 μm^2^) scanning area. The applied load is sufficient to displace the NTA molecules that are substituted by the T-OEG6 molecules present in the ethanol solution [65–66]. The nanografted areas have been then imaged in soft contact mode in ethanol or in saline buffer (Tris-HCl 20 mM, NaCl 150 mM, pH 7.4) with commercial cantilevers (CSC38 Mikromasch, *k* = 0.03 N/m) at the minimum force detectable (0.1 nN). Images and data were analyzed with XEI (Park Instruments) and Igor Pro (Wavemetrics Inc.) software.
